# Comparison of efficacy and safety of intravitreal ranibizumab and conbercept before vitrectomy in Chinese proliferative diabetic retinopathy patients: a prospective randomized controlled trial

**DOI:** 10.1186/s40662-022-00316-z

**Published:** 2022-12-01

**Authors:** Siying Li, Lili Guo, Pingping Zhou, Jiyang Tang, Zongyi Wang, Linqi Zhang, Mingwei Zhao, Jinfeng Qu

**Affiliations:** 1 Department of Ophthalmology, Peking University People’s Hospital, Beijing, China; 2 Eye Diseases and Optometry Institute, Beijing, China; 3 Beijing Key Laboratory of Diagnosis and Therapy of Retinal and Choroid Diseases, Beijing, China; 4 College of Optometry, Peking University Health Science Center, Beijing, China; 5 Department of Ophthalmology, The First Affiliated Hospital of Jiamusi University, Jiamusi City, Heilongjiang Province China

**Keywords:** Proliferative diabetic retinopathy, Anti-VEGF, Ranibizumab, Conbercept, Preoperative intravitreal injections, Diabetic vitrectomy, Efficacy, Safety

## Abstract

**Background:**

To compare the efficacy and safety of preoperative intravitreal injections of ranibizumab and conbercept in Chinese proliferative diabetic retinopathy (PDR) patients.

**Methods:**

This prospective randomized controlled trial enrolled 90 eyes of 80 patients with PDR. Forty-four eyes of 40 patients that received intravitreal ranibizumab (IVR) injections (0.5 mg/0.05 mL) before vitreous surgeries were assigned to the IVR group. Forty-six eyes of 40 patients that received intravitreal conbercept (IVC) injections (0.5 mg/0.05 mL) before vitreous surgeries were assigned to the IVC group. Intraoperative and postoperative indices were assessed for further comparison between the two groups.

**Results:**

There were no statistically significant differences in all surgery indices, including intraoperative indices (surgery time, *P* = 0.225; intraoperative bleeding, *P* = 0.808; endodiathermy use, *P* = 0.693; incidence of iatrogenic retinal breaks, *P* = 0.740; relaxing retinotomy, *P* = 0.682; retinal reattachment, *P* = 0.682 and silicone oil tamponade, *P* = 0.814) and postoperative indices (postoperative vitreous hemorrhage (VH), *P* = 0.808; neovascular glaucoma (NVG), *P* = 0.964; recurrent retinal detachment, *P* = 0.531; postoperative fibrovascular proliferation progression, *P* = 0.682 and reoperation, *P* = 0.955) between the two groups. There were no statistically significant differences in best-corrected visual acuity (BCVA) at each follow-up visit (*P* = 0.939, 0.669, 0.741 and 0.717, respectively) or in central retinal thickness (CRT) (*P* = 0.976, 0.699, 0.551 and 0.686, respectively). As for safety profile, both groups had no ocular or system adverse events during the observation period.

**Conclusions:**

IVR and IVC as a pretreatment of vitrectomy had similar efficacy and safety profile for Chinese PDR patients.

*Trial registration:* Registered at ClinicalTrials.gov (NCT05414149).

## Introduction

Proliferative diabetic retinopathy (PDR) is the most common cause of irreversible blindness in diabetic retinopathy (DR) [1, 2]. It is characterized by progressive loss of vision, diabetic macular edema (DME), vitreous hemorrhage (VH), retinal neovascularization, fibrovascular proliferation, tractional retinal detachment (TRD) and neovascular glaucoma (NVG) [3, 4]. Although pars plana vitrectomy (PPV) is the cornerstone for treatment of advanced PDR, related postoperative complications such as recurrent VH, NVG, and postoperative fibrovascular proliferation progression can still cause serious visual impairment. It is well known that vascular endothelial growth factor (VEGF) is an important agent for neovascularization, vascular permeability, and DME [5, 6]. Preoperative intravitreal injections of anti-VEGF drugs may represent a new strategy for making vitrectomy safer and more effective for severe PDR.

Currently, there are two types of anti-VEGF drugs available in China: (i) monoclonal antibodies such as bevacizumab and ranibizumab, and (ii) fusion proteins such as imported drug aflibercept and domestic drug conbercept. Monoclonal antibodies work by blocking only VEGF-A [7], but the fusion proteins act as a multi-target VEGF family blocker which can bind isoforms of VEGF-A, VEGF-B, and placenta growth factor (PlGF) [8]. Compared with aflibercept, conbercept has a different soluble receptor decoy (the fourth binding domain of VEGF receptor 2) which enhances the association rate of VEGF to the receptor [9]. There are studies comparing ranibizumab with aflibercept in age-related macular degeneration (AMD), DME, and pachychoroid neovasculopathy [8, 10, 11]. But studies comparing the efficacy of pre-vitrectomy intravitreal ranibizumab and conbercept, for patients with PDR are lacking [12–14]. Therefore, in this study, we designed a prospective randomized controlled trial to compare the efficacy and safety of preoperative intravitreal injections of ranibizumab and conbercept in Chinese PDR patients.

## Subjects and methods

### Patients

PDR patients with vitrectomy indication between September 2021 and February 2022 at Peking University People’s Hospital were screened for eligibility. The inclusion criteria were as follows: (a) patients aged 18 years or more with type 1 or 2 diabetes who were clinically diagnosed with PDR; hemoglobin A1c (HbA1c) ≤ 12%; (b) persistent VH for more than one month or recurrent VH with or without panretinal photocoagulation (PRP); (c) TRD detected by indirect ophthalmoscope or B-scan ultrasonography. Exclusion criteria included: (a) eyes had previous vitreoretinal surgeries (including vitrectomy or intravitreal drug injection); (b) eyes with any ocular disease that may hinder visual improvement other than PDR, such as optic atrophy or macular hole; (c) history of thromboembolic events (including cerebral vascular infarctions or myocardial infarctions) or coagulation system disorders or receiving anticoagulant or antiplatelet therapy; (d) eyes given gas tamponade or additional treatment like ranibizumab injection again or supplementary retinal photocoagulation during follow-up periods. The enrolled eyes were randomly assigned according to the Central Randomization System at a ratio of 1:1 to the IVR or IVC group.

### Intervention

Patients received 0.5 mg/0.05 mL IVR (ranibizumab, Lucentis; Novartis, Pharma AG, Switzerland) (IVR group) or IVC (conbercept, Chengdu Kanghong Biotech Co., Ltd., Sichuan, China) (IVC group) 3–5 days before three-port transconjunctival 25-gauge PPV. All patients underwent 25-gauge transconjunctival sutureless vitrectomy using the 25-gauge constellation system (Alcon, Fort Worth, TX, USA) under local or general anesthesia. A speed of 5000 cuts per minute was used for vitrectomy. Opacified vitreous fluid and fibrovascular tissues as well as blood clots adherent to the vitreous base were removed. Intraoperative hemostasis was obtained by increasing the infusion bottle height or endodiathermy. Intraoperative PRP was completed at the end of the surgery. Finally, an intraocular tamponade with silicone was applied if necessary. All surgical procedures were performed by the same experienced vitreoretinal specialist (QJF) who was blinded to patient allocation. All patients received antibiotics and 1% prednisolone eye drops with tapered frequency during the four-week period after surgery.

Baseline demographics and systematic conditions including diabetes mellitus (DM) and hypertension were recorded. All patients underwent a full ophthalmic examination, including automatic refractometry, best-corrected visual acuity (BCVA), intraocular pressure (IOP), slit lamp examination, indirect ophthalmoscopy, color fundus photography, spectral-domain optical coherence tomography (SD-OCT) (CIRRUS HD-OCT Model 5000, Carl Zeiss Meditec, Germany), ultra-wide field (UWF) fundus fluorescein angiography (FFA) using Optos 200Tx (Optos plc, Dunfermline, United Kingdom) and B-Scan ultrasonography if necessary. BCVA was analyzed on a logarithm of minimal angle of resolution (logMAR) scale; counting fingers vision was calculated as 0.01 (2.0 logMAR) and hand movement was calculated as 0.001 (3.0 logMAR). Central retinal thickness (CRT) was measured on the same machine using its tracking software known as FastTrac™ retinal-tracking technology for each follow-up visit [15]. The extent of vitreoretinal adhesion was defined following the previous classification system: absence of any adhesion was grade 0; focal adhesion less than of 3 sites was grade 1; broad adhesion of ≥ 1 site or adhesion at the disc, macular, or vascular arcade was grade 2; and vitreoretinal adhesion extending to the periphery was grade 3 [16]. Patients underwent all examinations at baseline, 1 day, 1 week, 1 month and 3 months after surgery.

### Statistical analysis

Baseline characteristics of all patients were collected and analyzed using SPSS Statistics 19.0 software (IBM SPSS Inc., Chicago, USA). Normally distributed continuous variables are presented as mean ± standard deviation (SD). Non-normally distributed continuous variables are presented as median (interquartile range (IQR)). Student’s t-test was used to compare normally distributed quantitative variables, while the nonparametric Wilcoxon signed rank test was used for non-normally distributed quantitative variables. Categorical data was analyzed using Pearson’s χ^2^ test or Fisher’s exact test. A threshold of *P* value < 0.05 was set for statistical significance.

## Results

### Baseline demographics

**Fig. 1:**
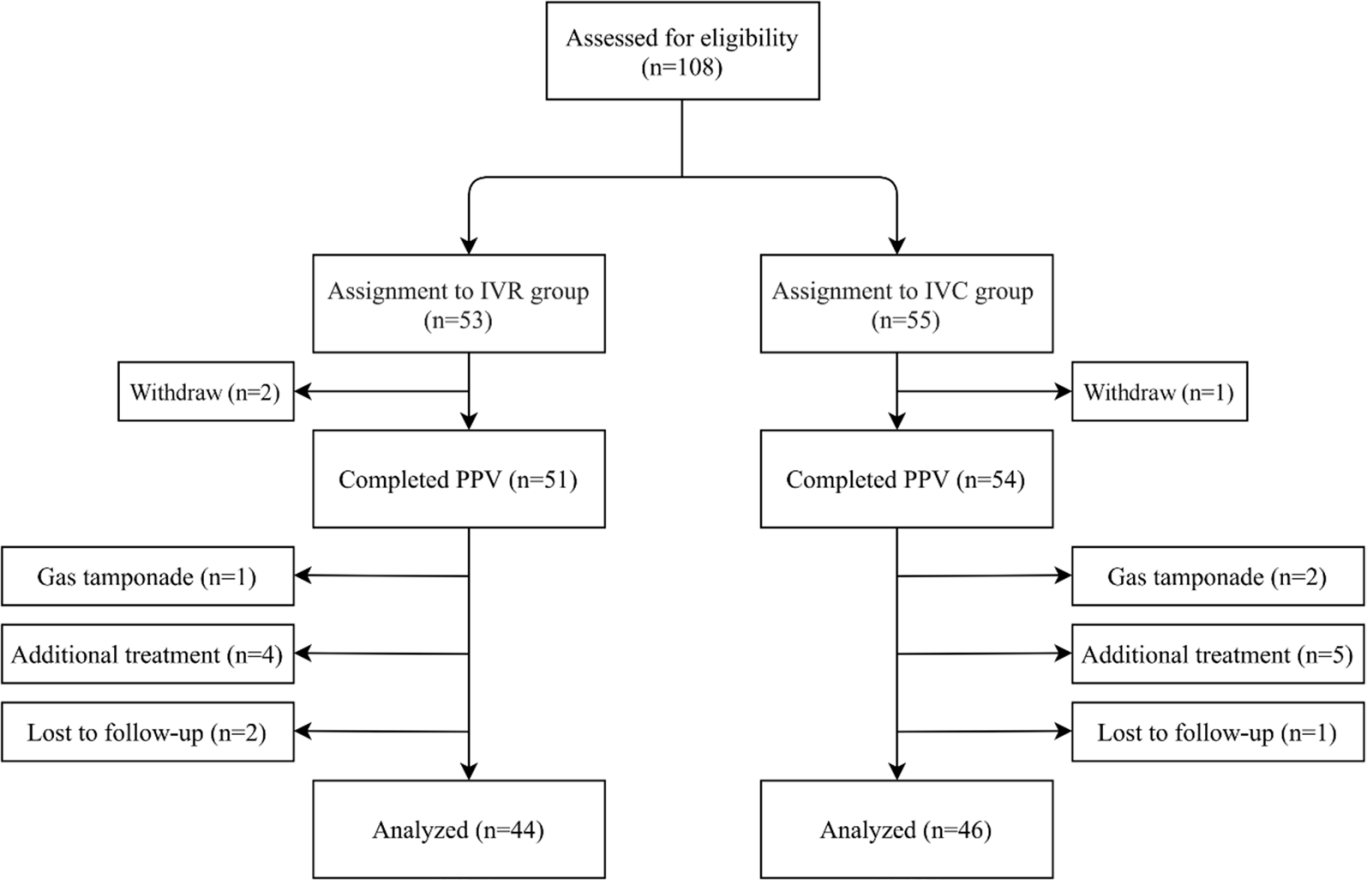
Flowchart showing the distribution of the study population. IVR, intravitreal ranibizumab; IVC, intravitreal conbercept; PPV, pars plana vitrectomy

A total of 108 eyes of 97 patients were enrolled in the study and assigned randomly to two groups: 53 eyes of 49 patients in the IVR group and 55 eyes of 48 patients in the IVC group. Three eyes withdrew (2 in IVR group and 1 in IVC group) before PPV. Fifteen eyes were removed from the protocol for gas tamponade during surgery (1 in IVR group and 2 in IVC group), additional treatment (4 in IVR group and 5 in IVC group) and lost to follow up (2 in IVR group and 1 in IVC group) during the 3-month follow-up period. Finally, 44 eyes of 40 patients in IVR group and 46 eyes of 40 patients in IVC group were included and analyzed in the study (Fig. 1). Baseline characteristics and demographics of all included patients in both groups are listed in Table 1. There were no statistically significant differences in age, sex, body mass index (BMI), type of diabetes, HbA1c level, duration of DM, hypertension, previous history of laser photocoagulation, status of lens, baseline BCVA, IOP, baseline CRT and extent of vitreoretinal adhesion grade (VAG) between the two groups (Table 1).

**Table 1:** Patient demographics

Characteristics	IVR group (n = 44)	IVC group (n = 46)	*P* value
Eyes/patients	44/40	46/40	–
Age (years)	54.50 ± 9.23	54.28 ± 10.61	0.920
Male/female	22/18	20/20	0.654
BMI (kg/m^2^)	24.31 ± 2.00	24.20 ± 1.94	0.814
Systemic profile			
Type of diabetes, n (%)			0.745
T1DM, n (%)	5 (12.5)	6 (15)	
T2DM, n (%)	35 (87.5)	34 (85)	
HbA1c (%), mean ± SD	7.03 ± 1.93	6.94 ± 1.71	0.831
Duration of DM (years), median (IQR)	8.50 (5.00, 11.00)	8.50 (5.00, 11.75)	0.693
Hypertension, n (%)	23 (57.5)	18 (45)	0.263
Ocular profile, n (%)			
History of laser	11 (25.0)	14 (30.4)	0.565
Pseudophakic	3 (6.8)	2 (4.3)	0.609
Indication for surgery, n (%)			0.821
Blurring VH	20 (45.5)	22 (47.8)	
TRD	13 (29.5)	15 (32.6)	
Diffuse FP	11 (25.0)	9 (19.6)	
BCVA (logMAR)	1.50 ± 0.70	1.51 ± 0.52	0.939
IOP (mmHg)	15.16 ± 3.09	14.67 ± 3.37	0.479
CRT (μm)	313.89 ± 122.97	314.61 ± 108.01	0.976
Extent of VAG, n (%)			
0	0 (0.0)	0 (0.0)	0.934
1	11 (25.0)	12 (26.1)	
2	19 (43.2)	21 (45.7)	
3	14 (31.8)	13 (28.3)	

*IVR* = intravitreal ranibizumab; *IVC* = intravitreal conbercept; *BMI* = body mass index; *T1DM* = type 1 diabetes mellitus; *T2DM* = type 2 diabetes mellitus; *HbA1c* = hemoglobin A1c; *SD* = standard deviation; *IQR* = interquartile range; *VH* = vitreous hemorrhage; *TRD* = tractional retinal detachment; *FP* = fibrovascular proliferation; *logMAR* = logarithm of minimum angle of resolution; *BCVA* = best-corrected visual acuity; *IOP* = intraocular pressure; *CRT* = central retinal thickness; *VAG* = vitreoretinal adhesion grade

### Surgical procedures and outcomes

The surgical procedures and outcomes are shown in Table 2. The average surgery time of the two groups were 61.11 ± 12.26 min and 64.13 ± 11.13 min, respectively. Intraoperative bleeding was defined as mild if stopped by increasing infusion pressure and/or by pressing with a blunt instrument, and severe if endodiathermy was required. The number of eyes with mild intraoperative bleeding was 5 (11.4%) in the IVR group and 6 (13.0%) in the IVC group, respectively. These two indices did not differ significantly between the two groups (*P* = 0.225 and *P* = 0.808, respectively, Table 2). Moreover, there were no significant differences in terms of endodiathermy use, iatrogenic retinal breaks, relaxing retinotomy, retinal reattachment and silicone oil tamponade between the two groups (*P* = 0.693, 0.740, 0.682, 0.682 and 0.814, respectively).

**Table 2:** Comparison of surgery indices

Surgery indices	IVR group (n = 44)	IVC group (n = 46)	*P* value
Surgery time (minutes)	61.11 ± 12.27	64.13 ± 11.13	0.225
Intraoperative bleeding, n (%)	5 (11.4)	6 (13.0)	0.808
Endodiathermy, n (%)	9 (20.5)	11 (23.9)	0.693
Iatrogenic retinal breaks, n (%)	3 (6.8)	4 (8.7)	0.740
Relaxing retinotomy, n (%)	2 (4.5)	3 (6.5)	0.682
Retinal reattachment, n (%)	42 (95.5)	43 (93.5)	0.682
Silicone oil tamponade, n (%)	19 (43.2)	21 (45.7)	0.814

*IVR* = intravitreal ranibizumab; *IVC* = intravitreal conbercept

Meanwhile, no significant differences were found in sum numbers of postoperative events at 3 months follow-up between the two groups (VH recurrence, *P* = 0.808; NVG occurrence, *P* = 0.964; recurrent retinal detachment, *P* = 0.531; postoperative fibrovascular proliferation progression, *P* = 0.682; and reoperation, *P* = 0.955; Table 3).

**Table 3:** Comparison of postoperative events

Postoperative indices	IVR group (n = 44)	IVC group (n = 46)	*P* value
VH recurrence, n (%)	5 (11.4)	6 (13.0)	0.808
NVG occurrence, n (%)	2 (4.5)	2 (4.3)	0.964
Recurrent retinal detachment, n (%)	2 (4.5)	1 (2.2)	0.531
Postoperative fibrovascular proliferation progression, n (%)	2 (4.5)	3 (6.5)	0.682
Reoperation, n (%)	3 (6.8)	3 (6.5)	0.955

*IVR* = intravitreal ranibizumab; *IVC* = intravitreal conbercept; *VH* = vitreous hemorrhage; *NVG* = neovascular glaucoma

BCVA improved during the follow-up in both groups (from 1.50 ± 0.70 to 0.95 ± 0.58 logMAR in the IVR group and from 1.51 ± 0.52 to 0.99 ± 0.48 logMAR in the IVC group). There were no statistical differences with respect to BCVA between the two groups at each point of follow-up visit (*P* = 0.939, 0.669, 0.741 and 0.717, respectively) (Table 4).

**Table 4:** Comparison of BCVA at each visit

BCVA (logMAR)	IVR group (n = 44)	IVC group (n = 46)	*P* value
Baseline	1.50 ± 0.70	1.51 ± 0.52	0.939
Postoperative 1 week	1.24 ± 0.63	1.29 ± 0.61	0.669
Postoperative 1 month	1.10 ± 0.63	1.14 ± 0.55	0.741
Postoperative 3 months	0.95 ± 0.58	0.99 ± 0.48	0.717

*logMAR* = logarithm of minimum angle of resolution; *BCVA* = best-corrected visual acuity; *IVR* = intravitreal ranibizumab; *IVC* = intravitreal conbercept

CRT decreased during the follow-up in both groups (from 313.89 ± 122.97 to 222.59 ± 36.52 μm in the IVR group and from 314.61 ± 108.01 to 225.63 ± 34.48 μm in the IVC group). There were no statistical differences in CRT between the two groups across every visit point (*P* = 0.976, 0.699, 0.551 and 0.686, respectively) (Table 5).

**Table 5:** Comparison of CRT at each visit

CRT (μm)	IVR group (n = 44)	IVC group (n = 46)	*P* value
Baseline	313.89 ± 122.97	314.61 ± 108.01	0.976
Postoperative 1 week	245.25 ± 61.98	250.09 ± 56.43	0.699
Postoperative 1 month	227.23 ± 42.37	232.39 ± 39.52	0.551
Postoperative 3 months	222.59 ± 36.52	225.63 ± 34.48	0.686

*CRT* = central retinal thickness; *IVR* = intravitreal ranibizumab; *IVC* = intravitreal conbercept

There were no ocular or system adverse events such as endophthalmitis, cardiovascular or cerebral vascular events in both groups.

## Discussion

PDR may be complicated with intraocular hemorrhage, fibrovascular proliferation and TRD, especially in eyes with an active neovascularization membrane. The surgical process could be quite difficult due to fibrovascular membrane hemorrhage, iatrogenic retinal breaks, extensive use of endodiathermy and retinotomy. Risks of recurrent VH, uncontrollable NVG, postoperative progressive fibrovascular proliferation, recurrent retinal detachment and reoperation rate is also high [17, 18]. VEGF has been proven to play an important role in neovascularization. Due to its immature vessel structure, bleeding and leakage in the retina usually occurs, leading to a thickened retina and poor vision. Intravitreal injection of anti-VEGF agents can induce vascular endothelial cell apoptosis and reverse the progress of retina neovascularization, resulting in abnormal vessel occlusion or regression as well as decreased fundus exudation and bleeding [19]. Moreover, it can also increase the number of pericytes, promote angiogenesis, as well as vascular maturation to maintain stability of the blood-retinal barrier [19]. Consequently, preoperative anti-VEGF injection could promote regression of neovascularization, reduce intraoperative and postoperative bleeding and thus improve the efficacy of surgery. However, one report demonstrated that injection for a long time (> 7 days) before surgery can increase the number of fibers and extent of proliferation as well as adhesion of the fibrous vascular membrane [20]. Hence, we chose to perform the injection 3–5 days before vitrectomy.

Among VEGF family members, VEGF-A plays an important role in vasculogenesis and neoangiogenesis, promoting cell proliferation, inhibiting apoptosis, increasing vascular permeability, vasodilatation, and recruiting inflammatory cells to the injury site. Therefore, it is the main triggering factor of pathological angiogenesis [21]. PlGF is a pleiotropic cytokine which binds to its membrane bound receptor fms related tyrosine kinase 1 (Flt-1) to stimulate angiogenesis [22]. Ranibizumab is a 48-kDa, specific, recombinant, humanized monoclonal antibody antigen-binding fragment with high binding affinity for VEGF-A, rendering it inactive in order to inhibit neovascularization and reduce exudation [23]. Conbercept is designed as a 141-kDa engineered fusion protein by gene recombination of the second immunoglobulin-like domain of VEGF receptor 1 and the third and fourth immunoglobulin-like domains of VEGF receptor 2 fused to the constant region (Fc) of human IgG1 [9]. It was demonstrated to stabilize the receptor-ligand complex and enhance dimerization [24]. The distinctive molecular design of this novel VEGF-trap mimics multiple human VEGFR domains, and hence, it can bind to all isoforms of VEGF-A, VEGF-B, VEGF-C, and PlGF with high affinity, precluding the activation of downstream signaling mediated by the VEGF family members [25]. Therefore, compared to ranibizumab, conbercept confers a wider range of targets and higher binding affinity. Its binding capacity to VEGF is 30 times that of ranibizumab [9]. In addition, there are different ways for the uptake of drug molecules. Ranibizumab penetrates the retina via intercellular clefts, whereas conbercept is taken up by ganglion cells, cells of the inner and outer retinal layers, and the retinal pigment epithelium [26, 27].

Previous studies have investigated the efficacy and safety of IVR and IVC for the treatment of patients with PDR, indicating that the two agents have similar efficacy [14, 28, 29]. However, they mostly used limited number of indicators in their comparison of drug efficacy [14, 28, 29]. In this study, we compared more outcomes including 7 intraoperative indices and 7 postoperative outcomes as indicators for comparison and found that there were no statistical significance in both efficacy and safety profiles between the IVR and IVC groups as pretreatment of vitrectomy for PDR patients. This result was consistent with previous studies. Cui et al. reported the efficacy and safety of the IVC, IVR, and intravitreal triamcinolone acetonide (IVTA) injection before PPV in patients with PDR and found that IVC and IVR could decrease difficulty in operation, enhance the success rate of PPV surgery, and reduce the incidence of postoperative complications better than IVTA, but there were no statistically significant differences between the two groups [14]. Yang et al. compared the efficacy of IVR and IVC as an adjuvant treatment before vitrectomy with silicone oil tamponade for PDR with TRD and found that they have similar efficacies for vitrectomy with silicone oil infusion in PDR [28]. On the other hand, some studies reported different outcomes. Lu et al. found that eyes treated with preoperative IVC had better visual outcomes than IVR for severe PDR [30]. One possible explanation for this inconsistency was the different sample sizes and inclusion criteria. Another possibility is the fact that inflammatory components are inevitably involved in the progress of PDR [31]. Conbercept was shown to not only reduce the level of VEGF but also significantly regulate cytokine networks, and reduce the protein levels of intercellular cell adhesion molecule-1 (ICAM-1), macrophage inflammatory protein-1 (MIP-1), IL-1β, IL-6 and TNF-α [32, 33]. Further investigations are still needed to draw a conclusion.

In addition, from the pharmacoeconomics point of view, conbercept was reported as a suitable and cost-effective option for the treatment of AMD and DME, compared with ranibizumab [34, 35]. Since the anti-VEGF agents will be washed out during the vitrectomy, a lower cost medication would also be a preferred choice if they have equal efficacy for severe PDR patients.

This study had several limitations. First, the sample size of this study was small. Secondly, a follow-up period of 3 months was relatively short. Thirdly, as a single-center study, the generalizability of the conclusion of this study has yet to be confirmed. Fourth, this was a study carried out in Chinese patients only, whether similar results can be found in other ethnics still need further investigation. Future studies with a larger sample size, longer observation period and multi-center participation will be needed to confirm the findings of this study.

## Conclusion

This study found that intravitreal injections of both ranibizumab and conbercept before vitrectomy had similar efficacy and safety profiles in Chinese patients with PDR.

## Data Availability

The datasets generated during and/or analyzed during the current study are available from the corresponding author upon reasonable request.

## Abbreviations

DR: Diabetic retinopathy
PDR: Proliferative diabetic retinopathy
IVR: Intravitreal ranibizumab
IVC: Intravitreal conbercept
IQR: Interquartile range
BCVA: Best-corrected visual acuity
VH: Vitreous hemorrhage
TRD: Tractional retinal detachment
NVG: Neovascular glaucoma
IVTA: Intravitreal triamcinolone acetonide
VEGF: Vascular endothelial growth factor
PPV: Pars plana vitrectomy
PRP: Panretinal photocoagulation
CRT: Central retinal thickness
